# Evaluation of Multi-level Social Learning for Sustainable Landscapes: Perspective of a Development Initiative in Bergslagen, Sweden

**DOI:** 10.1007/s13280-012-0378-y

**Published:** 2013-03-10

**Authors:** Robert Axelsson, Per Angelstam, Lennart Myhrman, Stefan Sädbom, Milis Ivarsson, Marine Elbakidze, Kenneth Andersson, Petr Cupa, Christian Diry, Frederic Doyon, Marcus K. Drotz, Arne Hjorth, Jan Olof Hermansson, Thomas Kullberg, F. Henry Lickers, Johanna McTaggart, Anders Olsson, Yurij Pautov, Lennart Svensson, Johan Törnblom

**Affiliations:** 1 Faculty of Forest Sciences, School for Forest Management, Swedish University of Agricultural Sciences, PO Box 43, 739 21 Skinnskatteberg, Sweden; 2 Faculty of Forest Sciences, School for Forest Management, Swedish University of Agricultural Sciences, PO Box 43, 730 91 Skinnskatteberg, Sweden; 3 Gamla Nåsvägen 10A, 770 10 Fredriksberg, Sweden; 4 LEADER Bergslagen, Foundation Säfsen Forests, Skinnskatteberg, Sweden; 5 Bergskraft Bergslagen Economic Association, Harald Olsgatan 1, 714 31 Kopparberg, Sweden; 6 Avjord Corporation, Leader Mellansjölandet, Vekhyttan Vreten, 716 93 Fjugesta, Sweden; 7 Swedish Forest Agency, Southern Dalarna District, 780 51 Dala-Järna, Sweden; 8 Lower Morava Biosphere Reserve, Narodnich hrdinu 23, 690 02 Breclav, Czech Republic; 9 Biosphärenpark Wienerwald Management GmbH, Norbertinumstraße 9, 3013 Tullnerbach, Austria; 10 Department of Nature Sciences, Institut des Sciences de la Forêt tempérée, Université du Québec en Outaouais, 58, rue principale, Ripon, QC J0V 1V0 Canada; 11 The Lake Vänern Museum of Natural and Cultural History (Vänermuseet), Framnäsvägen 2, 531 54 Lidköping, Sweden; 12 Skinnskatteberg Municipality, PO Box 101, 739 22 Skinnskatteberg, Sweden; 13 Ludvika Municipality, 771 82 Ludvika, Sweden; 14 Lekeberg Municipality, 716 81 Fjugesta, Sweden; 15 Akwesasne Task Force on the Environment, PO Box 992, Hogansburg, NY 13655 USA; 16 Biosfärkontoret, PO Box 77, 542 21 Mariestad, Sweden; 17 Teatermaskinen, Skräppbo Skola, 730 91 Riddarhyttan, Sweden; 18 Silver Taiga Foundation, PO Box 810, 167000 Syktyvkar, Komi Republic Russia; 19 Apel-FoU, Ånstagatan 6, 702 32 Örebro, Sweden

**Keywords:** Collaborative learning, Sustainable development, Case study, Knowledge production, Governance

## Abstract

To implement policies about sustainable landscapes and rural development necessitates social learning about states and trends of sustainability indicators, norms that define sustainability, and adaptive multi-level governance. We evaluate the extent to which social learning at multiple governance levels for sustainable landscapes occur in 18 local development initiatives in the network of Sustainable Bergslagen in Sweden. We mapped activities over time, and interviewed key actors in the network about social learning. While activities resulted in exchange of experiences and some local solutions, a major challenge was to secure systematic social learning and make new knowledge explicit at multiple levels. None of the development initiatives used a systematic approach to secure social learning, and sustainability assessments were not made systematically. We discuss how social learning can be improved, and how a learning network of development initiatives could be realized.

## Introduction

A multitude of international, national, and business policies describe a vision of sustainable natural resources use, human well-being, quality of life and rural development, as well as democratic governance (e.g., Aarhus Convention 1998; Council of Europe 2000; FAO 2003; European Commission 2004; Forest Europe 2011). The policy vision is thus sustainable landscapes including natural systems and space as well as human systems and place (see Haines-Young 2000; Antrop 2006). To implement such policies on the ground requires both achieving sustainability in the sense of satisfying economic, ecological, and social criteria (Montréal Process 2009; Forest Europe 2011), and sustainable development as societal steering processes at multiple levels of governance (Baker 2006). Resolving this dual challenge requires use of both “compass and gyroscope” (sensu Lee 1993).

The compass is about combining knowledge about the states and trends of sustainability indicators monitored with relevant verifier variables (Axelsson et al. 2013), and evidence-based or negotiated norms that define when sustainability has been achieved (Lammerts van Bueren and Blom 1997; Angelstam et al. 2013a). The gyroscope is about the need to adopt social learning (Leeuwis and Pyburn 2002; Keen et al. 2005) as an integral part of the policy implementation process. Practically, this implies systematic and active adaptive management and governance approaches, together with strong and competent project owners, process facilitators and collaborating stakeholders that view the implementation of policy as an experiment (Clark 2002). This includes the capacity to evaluate policies regarding their ambitions, to assess how well these ambitions are met by appropriate management in landscapes as integrated socio-ecological systems, input of expert knowledge into collaborative learning processes among stakeholders (Doyon et al. 2012), and bridging of the gap between researchers and the society/stakeholders (Palmer 2012).

In this context the term social learning describes a process where stakeholders collaboratively learn how to steer the development towards sustainability (Daniels and Walker 2001; Leeuwis and Pyburn 2002; Keen et al. 2005; Wals 2009). This process has been described as the combination of (1) reflections about experiences, values, ideas and the context for learning, (2) systems thinking to allow for a more holistic understanding, (3) integration of scales, world views, research disciplines, decision-making and synthesis, (4) negotiation and collaboration to handle conflicts and develop common ground, and (5) participation and engagement as a prerequisite for and to allow social learning (Keen et al. 2005; Dyball et al. 2009). Social learning thus includes an understanding of interdependencies (Bouwen and Taillieu 2004), learning about the places and their ecosystem services (Potschin and Haines-Young 2012), while at the same time the collaborative dimension is emphasized (Duff et al. 2009). In the context of social learning conflicts are often seen as an opportunity for change and learning (Folke et al. 2005; Schusler et al. 2010). A key challenge in social learning for sustainable landscapes is to move from local experiences and results to local tacit knowledge, and from tacit to explicit knowledge (Nonaka and Konno 1998; Brulin and Svensson 2012). Social learning is a sustainable outcome in any development project (Svensson et al. 2009). A collaborative learning process with stakeholders from different societal sectors and levels in social–ecological systems, or landscapes, need to consider issues like trust, norms, the interests of each stakeholder and the design and setting of the learning process (Habermas 1990; Ostrom 1990; Daniels and Walker 2001; Gray 2004; Sandström et al. 2011). A multi-level approach to social learning implies that there is a need to learn at all levels from local to international and to connect initiatives in different places to learn from each others’ experiences (Alppi and Åhlberg 2012; Angelstam et al. 2013b, c, d).

Natural resources such as forests, minerals, and waters have been of paramount importance for the socio-economic development in many countries (Angelstam et al. 2013d). Sweden is a good example. With increasing global demands there is an interest to further intensify the use of forests, increase prospecting of minerals, and increase wind-generated energy. Additionally, natural and cultural values are high-lighted as infrastructures for recreation and tourism (Vail and Hultkrantz 2000). As a consequence, several special initiatives aim at supporting development in rural regions. In Sweden, EU’s Leader concept is the Swedish government’s general approach to rural development (Moseley 2003). Additionally, landscape strategies are developed (Naturvårdsverket 2009), a network of Biosphere Reserves (BR) has been established (Elbakidze et al. 2013) and a suite of Model Forests (MF) have been proposed to support learning for sustainable landscapes.

The informal Bergslagen region in south-central Sweden illustrates these trends well (Ågren 1998; Angelstam et al. 2013d). With a long history as a strong industrial region based on natural resources, Bergslagen today suffers from a declining economy, performs poorer than surrounding areas (Andersson et al. 2012), and has been identified as a vulnerable area, with municipalities relying on one or a few industries only (Angelstam et al. 2013d). Sustainable Bergslagen is an initiative that has the ambition to unite different efforts by the development of multi-level collaboration and learning for sustainable landscapes in the Bergslagen region (see Table 1 in Andersson et al. 2012).

The aim of this study is to enhance social learning for sustainable landscapes by evaluating experiences from the local development initiative Sustainable Bergslagen and its network at local, regional, national, and international levels. This includes 18 local development initiatives that were used as a case study to explore the extent to which social learning takes place. First, we mapped the development towards multi-level collaboration in the Bergslagen region from 2000 to 2012. Second, we report and analyze practical experiences from all the development initiatives. The methodological framework was based on theories for collaboration, learning, and development. Finally, we discuss barriers and bridges for development initiatives to take the step towards becoming multi-level learning hubs for sustainable landscapes.

## Methodology

This study evaluates the multi-level social learning processes in Sustainable Bergslagen and its network using a transdisciplinary approach (Tress et al. 2006a, b; Hirsch Hadorn et al. 2008; Axelsson 2010). The two first authors of this article were elected as chairman of the board (R.A.) and secretary (P.A.) of the non-government organization Sustainable Bergslagen. Following the idea of transdisciplinary research the team of co-authors consists of researchers representing different disciplines, and practitioners from different societal sectors (Hirsch Hadorn et al. 2008). This team collaboratively developed a framework for this study, evaluated the 18 development initiatives, including Sustainable Bergslagen, and discussed the results. Official and informal meetings were documented by the two first authors, who also developed the text and connected it to relevant theories. This approach was complemented by participatory observations and numerous discussions with stakeholders in Bergslagen. Local co-authors commented, contributed by writing, reading, commenting and participating in discussions and finally confirmed that they agreed on the text. National and international level co-authors shared their knowledge during interviews and contributed by commenting and discussing the text.

First, to visualize the development of Sustainable Bergslagen towards multi-level social learning we mapped the development by listing projects, workshops and participation in meetings at multiple levels. These were attributed to the level of governance (local, the Bergslagen region, national Swedish, and international). Second, we evaluated how social learning for sustainable landscapes was approached in all 18 local development initiatives. For this we did not use any pre-defined model of what could be considered learning, but instead mapped any effort used with the aim of learning. Most of the studied initiatives belonged to four different concepts (sensu Axelsson et al. 2011), namely MF (IMFN 2008), BR (Elbakidze et al. 2013), EU Leader (Moseley 2003), Long-Term Socio-Ecological Research (LTSER) (Haberl et al. 2006), and two that were not designated to any concept (see Table 1). In the text we refer to these two as independent initiatives. The 18 development initiatives including Sustainable Bergslagen as a connecting hub were used as a case study of multi-level social learning (sensu Flyvbjerg 2011). This case study included, local, national and international initiatives that was a part of Sustainable Bergslagen’s network and Leader areas in and surrounding Bergslagen. In line with Flyvbjerg’s description of “most likely” cases, from the point of view of Sustainable Bergslagen, this suite represents a case study in which the authors knew that contacts and some collaboration between the initiatives were present, and where multi-level social learning thus likely would be present.

**Table 1:** The table shows all 18 development initiatives that forms this case study and that were used to study multi-level social learning (sensu Flyvbjerg 2011). The case study consists of the NGO Sustainable Bergslagen as a hub, its present network of other initiatives and initiatives in the Bergslagen area

Name	Concept	Country	Established
Eastern Ontario	Model Forest	Canada	1993
Lower Morava	Biosphere Reserve	Czech Republic	2003^a^
Vilhelmina	Model Forest	Sweden	2004
Wienerwald	Biosphere Reserve	Austria	2005
Kristianstads Vattenrike	Biosphere Reserve	Sweden	2005
Bergskraft	–	Sweden	2005
Komi	Model Forest	Russia	2006
Urbion	Model Forest	Spain	2007
Bergslagen	EU Leader	Sweden	2007
Mellansjölandet	EU Leader	Sweden	2007
Västra Mälardalen	EU Leader	Sweden	2007
Inlandet^b^	EU Leader	Sweden	2007
Gränslandet	EU Leader	Sweden	2007
Collectivité Forestière du Projet Le Bourdon	Model Forest	Canada	2008
Sustainable Bergslagen	–^c^	Sweden	2009
Vänerskärgården Kinnekulle	Biosphere Reserve	Sweden	2010
Bergslagen	LTSER	Sweden	2010
Vänern Landscape	LTSER	Sweden	2011

^a^The Lower Morava Biosphere Reserve was established in 1986 and took the step from a first generation to a second generation Biosphere Reserve when it was extended in 2003. Hence, 2003 is used as its year of establishment in this study

^b^Most of the Leader Inlandet area was designated as a Leader area named Våg 21 2001–2006

^c^Sustainable Bergslagen is listed as a MF candidate (http://www.imfn.net/index.php?q=node/159), i.e., not designated as MF

We defined a analytical framework including theories about (1) project ownership (sensu Brulin and Svensson 2012), (2) stakeholder/partner collaboration (sensu Arnstein 1969; Elbakidze et al. 2010), (3) knowledge production as production of new knowledge and as learning (Gibbons et al. 1994; Tress et al. 2006a, b; Axelsson 2010) leading to explicit knowledge (sensu Nonaka and Konno 1998), (4) results regarding both soft (process) and hard (on the ground results) (sensu Rauschmayer et al. 2009), and (5) networking (sensu Svensson et al. 2001; Senge 2006). These five criteria were also classified with respect to their level of governance from local, regional, and national to international (see Table 2). Data were collected through participatory observations, discussions with stakeholders in Bergslagen, and interviews with leaders of the development initiatives. A total of 285 activities were mapped (Table 1) and 18 interviews were made.

**Table 2:** Overview of the development concepts that 16 of the studied initiatives belong to and their main actors at different governance levels from a Swedish perspective. Bergskraft and Sustainable Bergslagen are not formally designated to any concept. Hence, we include the main sources of funding from the national and international level in the table. We use local and regional to express the geographical area of the development initiatives, which ranges from local landscapes, a municipality, to several municipalities or a region

	Model forest	Leader	Biosphere reserve	LTSER	Bergskraft	Sustainable Bergslagen
International	National Resources Canada/International MF Network	EU/The European Network for Rural Development	Man and the Biosphere Programme/EuroMAB	International and European ILTER Committees	EU funding	Funding to partners from EU, networking and development projects and transdisciplinary research at all three levels
National	Not present	National board of agriculture/Swedish Rural Network	Swedish Environmental Protection Agency/Swedish MAB Committee	Swedish ILTER Committee	Swedish Agency for Economic and Regional Growth	
Local and regional	Swedish and international MF initiatives	Swedish and international Leader initiatives	Swedish and international BR initiatives	Swedish and international LTSER initiatives	Local and regional partners and development programs	

Interviews were qualitative and open-ended (Kvale and Brinkman 2008). Each interview took 1–2 h. We used an interview guide that was based on the framework of this study. The interviews followed the guide but informants were given full freedom to express any opinion they had. The interviews were recorded and data related to the methodological framework were extracted from them into a data table. During the writing process we used an iterative model in which we went back and forth between the interviews and the text to confirm that the results were grounded in our data (Glasser and Strauss 1967).

## Results

### The Biography of Multi-level Collaboration in the Sustainable Bergslagen Initiative

**Fig. 1:**
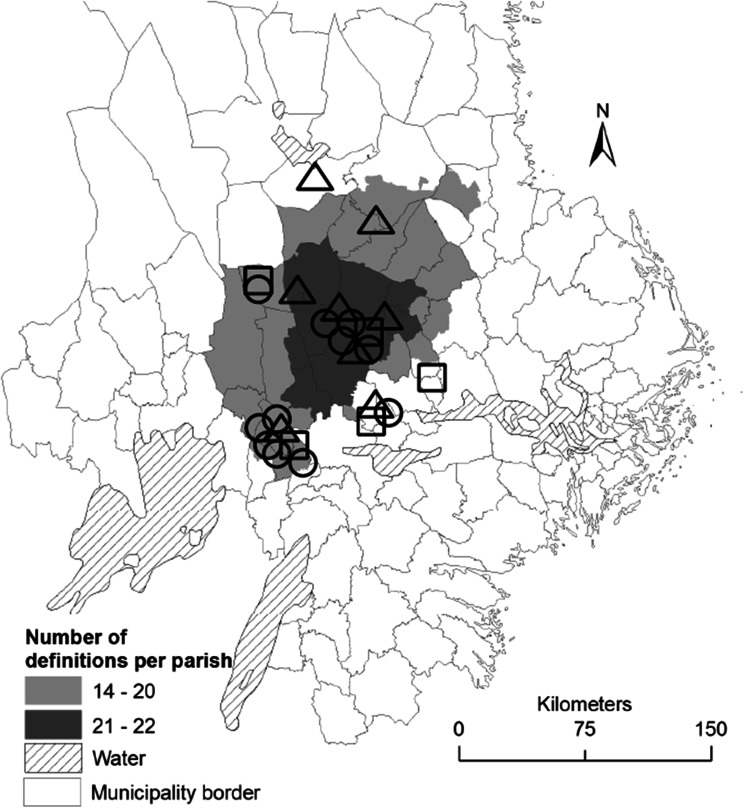
Map of Sustainable Bergslagen stakeholders located in the historical region of Bergslagen (Angelstam et al. 2013d) in south-central Sweden. *Circle* civil sector, *square* private sector, *triangle* public sector. Bergslagen is in this figure shown as areas that match multiple Bergslagen definitions following Andersson et al. (2012)

The development initiative Sustainable Bergslagen began with the Foundation Säfsen Forests in 2000 (for details see Angelstam and Törnblom 2005; Elbakidze et al. 2010; Table 1 in Andersson et al. 2012). Since then it has developed into an emerging network of stakeholders and clusters and covers the whole Bergslagen region (Andersson et al. 2012; Angelstam et al. 2013d; Fig. 1). Sustainable Bergslagen is a network of mainly local and regional stakeholders inspired by general principles for sustainable development and sustainability, such as the ecosystem approach, landscape approach (Axelsson et al. 2011), sustainable forest management (FAO 2003), scholarly work such as adaptive governance and multi-level governance, and different development concepts including but not limited to MF and BR. In 2009 Sustainable Bergslagen was formalized as a non-government organization (Fig. 1). According to its statutes: “The society Sustainable Bergslagen is a platform and a network for co-operation between different actors and natural resource users, that through thinking, innovations and knowledge production wish to contribute to sustainable development, rural development and a living landscape in Bergslagen. We want to be active from idea to implementation.”

**Fig. 2:**
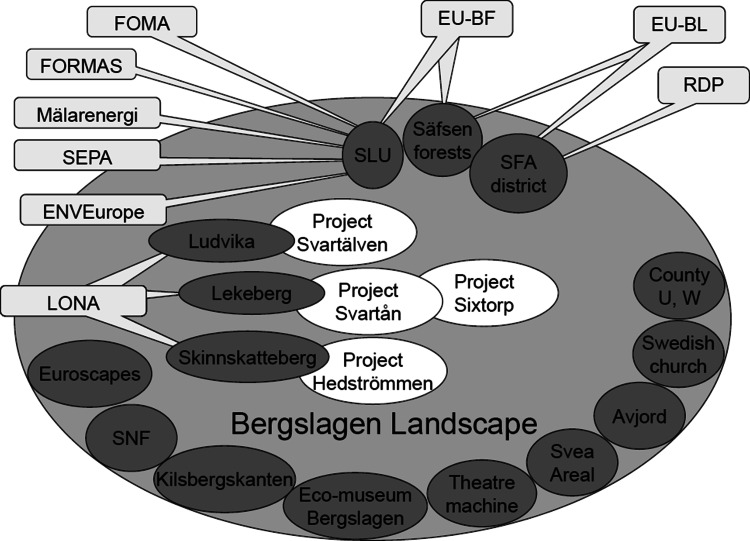
Illustration of the organization and funding of the development initiative Sustainable Bergslagen, including different stakeholders (*gray ovals*), and examples of their contribution in kind or by projects (*white ovals*). In the *upper part* of the figure it is shown how partners have different sources of funding for their activities and participation (*light gray boxes*). An example is Lekeberg Municipality which funds the projects Svartån and Sixtorp

**Fig. 3:**
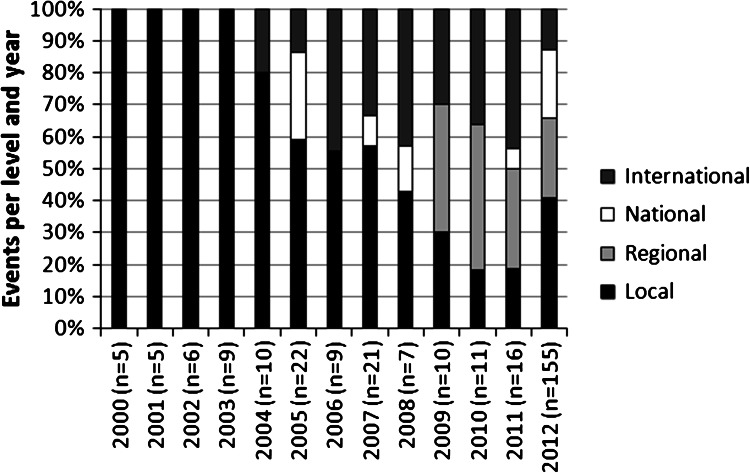
The proportion of local, regional, national, and international activities such as projects, meetings, and other important events from year 2000 to present for Sustainable Bergslagen (including its origin from the Foundation Säfsen Forests; see Elbakidze et al. 2010). During this time period the number of reporting stakeholder groups has increased from one to five

The participants in the learning process contribute with their own funding and time, and some have been successful in bringing in external funding both for individual projects, and to support collaboration (Fig. 2). The transition from local collaboration to a multi-level network for social learning took a long time and is still a process in development (Fig. 3). The absence of basic funding made stakeholders contribute in kind, and initiated activities inspired by the collaboration. This made the development a slow but organic process (Fig. 3). After two rounds of applications for funding of multi-level collaboration, a key opportunity was the participation in the Baltic Forest project 2006–07 funded by EU InterReg with about 25 partners from eight countries in the Baltic Sea Region. The main aim of the project was to establish local collaboration and a network of forest landscapes to promote rural development inspired by the MF concept. As a part of this project it was possible to work actively at the local level in Säfsen, in the Bergslagen region, with other initiatives in Sweden, and at the international level (see Table 1).

### Experiences from Social Learning

#### Project Ownership

Funding and host organizations, i.e., project owners must know what they want and use the project to reach their own and the society’s policy visions. Of the 18 development initiatives a clear majority was to some extent steered by donors and funding programs. MFs in Canada were funded federally, the Spanish MF had mainly regional funding, while the Russian MF was funded from abroad, and the Swedish MF had short-term intermittent funding for some of its projects. As an EU Member, Sweden is using the EU Leader method (Moseley 2003) for rural development. The Leader programs have joint national, municipal, and EU funding. For BRs in Sweden and Czech Republic there was some basic government funding, even if the studied BR in Czech Republic did not receive this kind of funding. It was instead funded by its founding organizations and different projects. Sustainable Bergslagen had no basic funding, and was instead funded by in kind and project contributions from stakeholders (see Fig. 2). Finally, one of the independent initiatives started as local initiative to promote rural development in one municipality and today work to support sustainable mining in the Bergslagen region funded by the EU, regional administrations, and member municipalities. Additionally, the Russian MF and one of the independent initiatives earned parts of their funding from selling training, courses and services related to natural resource use and management. For a well-developed partnership or local stakeholder collaboration to be able to steer their own and their area’s development in a desired direction it is important to not be dependent on one kind of funding. One alternative model is for partners to bring in their own funding for the collaboration and to deal with power relations internally.

Most development initiatives reported that they experienced weak project ownership, and a lack of competent project donors that knew what they wanted. For one of the independent initiatives a representative of the project owner, a municipality, was a key part of the project management and leadership. Still, very few of the organizations that provided funding for the development initiatives had skills to support, steer, and facilitate their development as hubs for collaborative learning. The local development processes instead had to follow strict regulations connected to the funding bodies’ administrative procedures. Some of the initiatives claimed that they could probably get technical support, such as GIS support, help with inventories and different kind of technical analyses if requested. Some initiatives also got this kind of technical support from project partners.

#### Collaboration

As formal organizations or as designated to specific concepts, 15 of the 18 studied initiatives were 1–7 years old (Table 1). Nevertheless, about half of them were built on more than 10 years of local collaboration. Most had passed a starting point, where stakeholders agreed to collaborate, they had prepared for their work and learnt about their area mainly through the process of writing their application (for funding or to become designated with their respective concept). A clear majority had not done any comprehensive external or internal evaluations or reflections, including critical learning to adapt their work and development to better steer towards their goals. One BR did an internal evaluation of their activities together with their founders regularly. Similarly, the oldest MF regularly arranged a retreat where mainly the management reflected over activities, results, and their vision. They reflected using traditional knowledge methodology for collaboration, including reflections about respect, equity, and empowerment among their partners (see Story and Lickers 1997; Holmes et al. 2002).

Only one of the independent initiatives used or continuous evaluations done by external consultants/researchers to support learning and development as this was required by their funding program. In a few cases project level self-evaluations and external evaluations were used. Most of the initiatives had started to consider the need for evaluations, reflections, and critical learning to learn how to improve their work. However, they were not sure about how to proceed with this endeavor.

Within their own concept networks most initiatives were in a phase where they learned to know each other, and exchanged experiences when they met, most often at network level meetings. Two initiatives also participated in several concept network level projects as well as coordinated and planned network level meetings, and for one initiative this networking had been evaluated. This initiative mentioned that they were part of a regional network that was well funded for several years. As a consequence, evaluations and reflections resulted in critical learning, adaptations of networking and local activities. However, several mentioned the lack of common projects among initiatives in the respective development concept networks as a problem.

Most of the initiatives had developed some level of collaboration at the local level with public, civil, and business sectors represented. The exceptions included a few initiatives where the civil sector was absent and one where the aim was to create research collaboration in the area, and where the interface towards the society had not yet been addressed. At higher governance levels, the development initiatives’ collaboration was less developed. Often some governmental organizations or regional NGOs were mentioned as partners. At the national level, universities, government agencies, and NGOs were mentioned as partners. For the international level no specific actors were mentioned, but most of the initiatives took part in international meetings connected to their concept and international projects. In addition the networks of the studied development initiatives consisted mainly of their concept networks (i.e. MF, BR, and EU Leader, LTSER) at national, regional, and international levels. Almost all initiatives consisted of an organization with members that elected a board of representatives. The board then hired an operative function. In a few cases the board also cared for the operative function. The organization of the studied initiatives ranged from formal meetings, to voluntary associations, non-profit associations or social enterprises, and foundation. For one of the independent initiatives, a carefully constructed group of organizations, consisting of an economic association and two corporations had been created. In addition there were often advisory groups or working groups where the board or the operative function could interact and get advice from stakeholders representing different interest areas. In one initiative the main organization was a public benefit company with founders and a management board that had also non-founder members. With other stakeholders they had a collaborative relationship, working together in joint projects. The local champions (sensu Jones 2002) of the initiatives ranged from local civil sector groups, regional administrations, and local government units. Often municipalities participated early in the establishment of an initiative, and played an important role in the development of the initiative.

#### Joint Knowledge Production

All 18 initiatives operated and reported projects, and tried to disseminate their results. Projects resulted in a vast array of experiences locally. There were, however, few activities aiming to produce first tacit and then explicit new knowledge from their local experiences. The initiatives did not use any kind of systematic approach with the aim to secure learning and adapt activities, even if in a few cases an individual partner did that. Instead the aim of joint projects and activities was to produce a solution to a local problem. In a few cases, researchers did consultancy work for the initiative. In other places researchers did research in the designated area, but reported or published their results independently, and sometimes the results were communicated in popular publications. In a few of the cases the local champion felt like the initiative was a study object of several researchers. The initiative supported the research with their participation, but rarely felt that they got any feedback in return or help to solve local problems. In another case a new project was just launched where the initiative would work in an integrated fashion with researchers from different research disciplines and a group of stakeholders to solve an urgent issue. It was also clear that for many of the non-academic local champions the border between reports, reports written by researchers, books, peer-reviewed book, and peer-reviewed articles was not clear and fully understood. In one of the independent initiatives the academic partner had steered its research, during an early phase of the collaboration, towards knowledge production and research about the region and its sustainability status and development. This was then used as a basis for the further development of collaboration.

The results from projects were generally presented as written reports aimed to satisfy the donor. In one initiative they presented results in reports and in addition published bi- or trilingual popular books. Another initiative wrote booklets and reports, and arranged training to disseminate their results. They had reached large numbers of stakeholders in their part of the country and even from neighboring countries. In addition to reports required by the donors, the two independent initiatives, including their partners, wrote mainly scientific articles. The reports were sometimes written by a single stakeholder or partner and sometimes co-authored by a group of project partners.

In general the 18 initiatives had neither any approach to critically reviewing its activities and results from projects with the aim to promote social learning locally or regionally, nor compared them with results from other areas or relevant research. In a few initiatives, projects and results were regularly critically examined, more like an audit, together with project donors to control whether the money spent yielded the desired results. In only one of the initiatives they used external reviewers, even if several of the others saw the benefit of it; one initiative was working actively to set up a system of peer-review within their concept network. Within individual projects, however, self-evaluations and reflections were used as tools to adapt and learn with the aim to steer the project. For most initiatives the development of their strategy was an initial process, and only a few of the initiatives continuously adapted their strategy. Instead the strategy was often openly written to allow for changes in direction.

#### Results of the Development Initiatives

All initiatives claimed to have soft process-related results, such as increased social capital (sensu Lin et al. 2001; Axelsson et al. 2013) and an improved capacity to collaborate. They also mentioned that process-related results were harder for them to report and demonstrate. Two initiatives had been very successful in working with schools to encourage student interest in natural science and natural resource management by involving them in monitoring and studies of polluted sites. Most initiatives also claimed hard results. These included improved business opportunities, increased income for local companies, stakeholder participation in study programs, and improvements of technical and green infrastructures. Other examples included construction of bathing facilities in lakes to restoration of polluted areas after old industries abandoned them, to the introduction of a new model for public hearings related to natural resource management used by a government agency. Two initiatives mentioned that their work had brought their topics up for discussion, made the topics visible in media and thus had resulted in some learning among stakeholders in general. The local level was very important for all of the studied initiatives. Without early noticeable results locally for individual stakeholders it was often hard to attract local people to participate.

#### Networking

Networking was described as going to meetings and conferences, listening to presentations and meeting people from other places. All informants were convinced of the need for networking as a way of learning. They indicated that this had often been very valuable and rewarding, and that they had learnt a lot. Many of the informants also expressed that it was hard to get support from project owners and local stakeholders for networking and national or international collaboration. There were no or very few attempts toward structured social learning at the network level. The lack of common projects in the networks was also mentioned as a problem. Two of the studied initiatives had initiated collaboration based on how to use a similar approach to learn about their own area, including its history, land cover, land use, status and trends of the social and ecological systems.

## Discussion

### The Challenge of Joint Collaborative Learning at Multiple Levels

There are many approaches to learning. Brulin and Svensson (2012) proposed learning through continuous evaluations and structured reflections as a way for development projects to learn for joint actions and adaptations. This study shows that development of the studied initiatives was iterative stepwise through the four phases (starting, preparatory, implementation, and evaluation). Sustainable Bergslagen and most of the other development initiatives, were in a phase of implementation and operation of projects. The focus was to produce local results, and to anchor the work locally. At the network level the main activity was to meet and discuss experiences. Since most of the initiatives were young, they had not reached the evaluation phase yet. However, it was clear that older initiatives (Table 1) thought more about evaluations as a tool for learning, even if this had most often not been implemented yet. These experiences are consistent with other studies showing that local development processes take time (Borrini-Feyerabend et al. 2004; Blagovidov et al. 2006; Tress et al. 2006a, b). Another explanation could be that evaluations are often seen as something negative, i.e., someone that controls that you have done your job well and where learning to support the initiative or project is not the main aim (Svensson et al. 2009). Several of the informants also claimed that it is hard to get local support for national and international collaboration, because people are rooted in their local areas and regions. Thus many regard national and international levels as more abstract and difficult to understand (Escobar 2001).

Several major challenges for a development initiative were identified. The first was to develop general understanding among partners that steering of the society includes different sectors at multiple governance levels (Bache and Flinders 2004). The second was to find ways to work with all land owner categories in an area (see also Richnau et al. 2013), and that many stakeholders were not committed to collaboration. The third was to assess the consistency among policy documents from different sectors and levels. The fourth was to have access to transparent and reliable data about the states and trends of landscapes’ different dimensions of sustainability, and to connect this to the initiative’s place. The fifth was to understand where and how decisions are made and how to influence decisions at multiple levels in society. Finally, the creation of capacity to cope with these challenges is an important defense against becoming a marginalized rural area (Persson and Westholm 1994; Commins 2004), especially in regions with a negative development. Hence, to avoid or reduce social exclusion (Slee 2002), there is a need to have representatives from the initiative who can follow political decisions, policies and regulations at multiple levels of governance, and who will react and act when needed.

We conclude that the main barriers to joint collaborative learning among different stakeholder categories were the following:

(1) Public sector organizations with a responsibility to lead, secure or facilitate the sustainable development process, including more specific issues like regional development, often have problems to address issues in an integrated way and to collaborate as equals with stakeholders. Public organizations will thus not be able to solve the tasks without learning how to collaborate with other sectors.

(2) Civil sector stakeholders often have claims related to the realization of sustainable landscapes. For them there is a need to develop good relations with other sectors, to collaborate and thus to ensure the achievement of their goals. They often have a problem with competence, and to participate in collaborative learning, even within their own area.

(3) Private sector businesses that use natural resources are steered by owners’ economic ambitions, and are regulated by societal policies about sustainable resource use. By taking an active part in local and regional level sustainable development processes, they can contribute in a constructive and positive way, and may not be caught by surprise when norms and values change (Lee 1993).

(4) In general, stakeholders often do not see sustainable development as a societal process, where stakeholders from different sectors learn together at multiple levels (i.e., social learning) to steer the development towards a desired goal.

(5) Finally, there is a general need for better knowledge and understanding of sustainability policies, the present sustainability status and development trends of the landscape or area in focus. This often requires an improved collaboration with researchers and researchers who are truly interested in stakeholder collaboration and to contribute to the sustainable development process, for example, by providing data about sustainability status and trends.

### Towards Structured and Joint Multi-level Learning

In general, learning takes place when learners can relate their studies as well as written and presented material to their own experiences, and critically discuss the validity of this information in a group where they feel safe and comfortable (Ramsden 1992). In groups with adult participants, outside a formalized school setting, the latter is even more important (Kolb 1984; Vella 2002). Hence, collaboration needs to be built on respect, equity and empowerment (Story and Lickers 1997; Gray 2008) that will create a space for learning (Lattanzi 1998; Nowotny 1999; Nowotny et al. 2001). The term “reflecting practitioner” captures this (Schön 1983; Clark 2002), meaning to understand policies, to experiment, to critically assess, and to reflect on one’s own activities with the aim to do a good job.

Related to multi-level social learning, there are three important parts. First, there is the local-level process, where projects develop solutions to different problems. Second, there is learning from these local experiences. The third part is the general learning based on experiences from multiple development initiatives and places (Angelstam et al. 2013b), and where tacit knowledge is generalized to become explicit (Nonaka and Konno 1998). The production of new knowledge is characterized by both the new knowledge itself and that this new knowledge is used (Gibbons et al. 1994). This kind of collaborative learning (Daniels and Walker 2001; Gray 2008) takes place when project results are assessed, when stakeholders try to understand why it worked, what kind of problems there were, where it could have failed and relates it to their own experiences, i.e., to discuss success factors, failures and to reflect on the projects and the results (Svensson et al. 2009). Learning processes will benefit from a transdisciplinary approach (Naveh 2007; Hirsch Hadorn et al. 2008; Angelstam et al. 2013e), which includes analysis of the collaborative learning process (Daniels and Walker 2001; Svensson et al. 2009) and compares results to theories and experiences in other places (Starrin et al. 1991). This contributes to socially robust results (Nowotny 1999; Nowotny et al. 2001; Svensson et al. 2009) or sustainable knowledge (Gustavsson 2000). It is, however, important to see difference between socially robust solutions and solutions that simply do not affect the power relations among stakeholders.

To achieve social learning there is often the need for a neutral facilitator that helps non-academic and academic stakeholders through this process of transdisciplinary research (Daniels and Walker 2001). Learning and knowledge production will benefit if the stakeholder group includes different sectors and levels, different interests, and if people have different experiences and backgrounds (Brulin and Svensson 2012). This process of learning in a local development initiative is complex, and requires that people with different skills contribute and that stakeholders are open-minded and willing to participate in the learning process. Since one important part of the knowledge production process is learning among the participating stakeholders, the importance of relevant stakeholder representation cannot be overemphasized (Brulin and Svensson 2012).

Bringing this process of collaborative learning to the network level (concept networks and other networks) will increase the complexity. This is associated with several additional challenges. Activities with the aim to enhance learning, like reflections, discussions and self-evaluation need to be complemented with external assessments of projects and the local initiatives as input to a collaborative learning process in the network. The specific challenges at the network level including: (1) the abstraction of the learning process which risk losing contact with reality and local stakeholders (Escobar 2001); (2) the homogeneity of participants (i.e., mainly leaders or champions of development initiatives participate in network level meetings); (3) the absence of attempts to build trust among development initiatives to enhance collaboration; and (4) the limited equity among initiatives, government representatives and politics. As within a local development initiative it is probably wise to learn collaboration at multiple levels (Table 2). First, collaboration should focus on solving small and easy problems before taking on the bigger questions (Story and Lickers 1997). Comparative studies using the same analytic framework in different places (Ostrom 2009; Angelstam et al. 2011, 2013b, e) will provide important input to the learning process (e.g., Svensson et al. 2009; Elbakidze et al. 2010).

This study revealed several gaps related to how development initiatives’ experiences and projects contribute to social learning and how local or tacit knowledge is further processed towards general or explicit knowledge at multiple levels. We see a clear need for further studies that use other sources of knowledge than the studied initiative’s experiences about how to accomplish multi-level social learning.
